# Case study reflecting principles and treatment techniques of postural restoration

**DOI:** 10.1186/1748-7161-9-S1-P7

**Published:** 2014-12-04

**Authors:** Susan Henning

**Affiliations:** 1 Advance Physical Therapy, Chapel Hill, NC, USA

## 

The etiology of scoliosis has eluded definition. Relative Anterior Spinal Overgrowth (RASO) is generally accepted as an initiating phenomenon but predisposing factors to RASO are not understood.

A revolutionary new approach to physical therapy known as Postural Restoration (PR) which, in the US, has been especially successful in sports performance enhancement, offers a perspective on human bio-mechanics which may help clarify predisposition to scoliosis. Additionally, PR treatment techniques offer powerful tools to balance asymmetrical function. The case study demonstrates the effectiveness of early intervention. Clinically, PR has also proven successful in rehabilitation for older adolescents and for adults.

PR is the first approach to appreciate the significance of fundamental asymmetry of the human body as a positive factor, facilitating movement via a universal right side dominant movement pattern. This pattern represents one half of the gait cycle; its opposite pattern, on the left, is less used and less strong.

Right side dominance manifests in a tri-planar system. PR recognizes the essential role respiratory function plays in the bio- mechanical organization of the body. Breathing discord effects changes in diaphragm form and function causing it to lose respiratory effectiveness and to assume a more structural role, which then reinforces lordosis. As a result, shear forces on dorsal vertebral growth plates result in RASO as described by the Heuter-Volkman principle.

RASO obstructs normal thoracic flexion. Compensatory strategies to achieve flexion, which follow dominant right side patterning will demonstrate a thoracic dextro-scoliosis, with or without a compensating lumbar levo-scoliosis.

PR rehabilitation begins with sagittal plane balancing. Activities follow which balance frontal plane asymmetries by inhibition of dominant muscle chains and facilitation of the non-dominant pattern. Transverse plane activity is integrated once sagittal and frontal plane relative balance has been achieved. Programs usually begin in non-weight bearing positions and progress to upright, then to alternating reciprocal movement. ADL activities are modified. All exercises rely on respiratory coordination.

This case study follows a 9 year old, hypermobile girl, diagnosed by x-ray with a right thoracolumbar curvature of 27 degrees, who was able to eliminate her curvature, by x-ray, over a three month period with PR.

## Consent

Written informed consent was obtained from the parents/legal guardian of the patient for publication of this Case report. A copy of the written consent is available for review by the Editor of this journal.

